# Supplementary material for: Identification of the GDP-L-Galactose Phosphorylase Gene as a Candidate for the Regulation of Ascorbic Acid Content in Fruits of Capsicum annuum L
Source: Int J Mol Sci. 2023 Apr 19;24(8):7529. doi: 10.3390/ijms24087529 (PMC10145300; doi:10.3390/ijms24087529)
Supplement: Supplementary file 1 [file ijms-24-07529-s001.zip › Table S2.pdf]

Table S2: Summary of RNA-Seq library information.

Sample	Total reads (million)	Q20(%)	Q30(%)	GC content	Total mapped (million)
Z6_day 10	76.13±0.59	99.11±0.02	96.58±0.06	43.50±0.13	72.30±0.56
Z6_day 50	74.70±1.46	99.09±0.04	96.52±0.15	43.47±0.05	70.90±1.53
Z6_day 80	60.77±3.91	99.10±0.05	96.58±0.21	44.46±2.34	52.65±4.93
Z5_day 10	73.09±3.37	99.10±0.03	96.55±0.06	43.34±0.12	62.49±3.38
Z5_day 50	75.04±6.02	99.10±0.02	96.56±0.06	43.58±0.22	65.58±5.18
Z5_day 80	70.00±1.60	99.10±0.11	96.55±0.34	43.47±0.35	60.47±2.01
