# Supplementary material for: Identification of the GDP-L-Galactose Phosphorylase Gene as a Candidate for the Regulation of Ascorbic Acid Content in Fruits of Capsicum annuum L
Source: Int J Mol Sci. 2023 Apr 19;24(8):7529. doi: 10.3390/ijms24087529 (PMC10145300; doi:10.3390/ijms24087529)
Supplement: Supplementary file 1 [file ijms-24-07529-s001.zip › Table S6.pdf]

Table S6: Primers used for VIGS construction and detection.

Gene name	Gene symbol	Sequence
CA.PGAv.1.6.scaffold919.38	CCS	F: TTCCAAATCCAACCAAACAAAA
		R: GGGTCAACGCAACATACCTTAA
CA.PGAv1.1.6.scaffold65.175	GGP	F: ATGATGCTTAAGATTAAGAGGGTTCC
		R: TCTGCTGACGATCCTCCCAT
	TRV2	F: TGTTACTCAAGGAAGCACGAT
		R: CCTAAAACTTCAGACACGGA
	TRV1	F: GAAAATATTGCTGCGCCTAACG
		R: ACCTGCCACGGTTCGAAGTA
