# Supplementary material for: Identification of the GDP-L-Galactose Phosphorylase Gene as a Candidate for the Regulation of Ascorbic Acid Content in Fruits of Capsicum annuum L
Source: Int J Mol Sci. 2023 Apr 19;24(8):7529. doi: 10.3390/ijms24087529 (PMC10145300; doi:10.3390/ijms24087529)
Supplement: Supplementary file 1 [file ijms-24-07529-s001.zip › Table S7.pdf]

Table S7: Primers used for the qRT-PCR validation.

Gene name	Amplicon size	Sequence
CA.PGAv.1.6.scaffold421.5	236 bp	F: AAATCTTCGGTTACTGATATTGGAC
		R: ATACTCTTACCAGACAGAAACCTCG
CA.PGAv.1.6.scaffold2122.5	292 bp	F: CATCCAACTATTGATTTCCCTGGC
		R: TGTCCAACGGAACATCAGTTAGAT
CA.PGAv.1.6.scaffold134.77	215 bp	F: ACGAAGATGTTGTCGAGGTTTT
		R: GCAGTTAATGATTTCTGTGCTG
CA.PGAv.1.6.scaffold1777.1	289 bp	F: TTTCATTCTACCTCCCTCCAACC
		R: TACCATCTCAGCCATGTCCTCGT
CA.PGAv.1.6.scaffold6.15	212 bp	F: CCGAAAGTGAGAAGTATGGATTG
		R: GCTTGAGTGTGTTACAGGAGAACG
CA.PGAv.1.6.scaffold291.8	214 bp	F: GTGAGACAGAGACCGTGGGGAA
		R: GAGGTGAAGTATCTTGAGAAAGCAA
CA.PGAv.1.6.scaffold490.14	124 bp	F: GAGAGCAGAACCCCAACATCA
		R: AAAACACCAGGAACGAAACCA
CA.PGAv.1.6.scaffold767.43	274 bp	F: AAACTTTGTACTATGGAAGGAGC
		R: GAGGTGTTGTAACACTAGACATG
CA.PGAv.1.6.scaffold1034.48	232 bp	F: TCTCTTCTTCGCTTCAAATGTGTCT
		R: CATAGTGGGTTAAAGGGGGATC
CA.PGAv.1.6.scaffold1234.3	152 bp	F: GAAGGAAGGCCATGGGGTCAT
		R: GCAACAAAGCTGTGGAATAGAAC
UBI-3	216 bp	F: CACATGTACAAGACTGACAGGGCCA
		R: AGACCCGTTCCTTGACAACCCAC
