# Supplementary figures and images for: Identification of the GDP-L-Galactose Phosphorylase Gene as a Candidate for the Regulation of Ascorbic Acid Content in Fruits of Capsicum annuum L
Source: Int J Mol Sci. 2023 Apr 19;24(8):7529. doi: 10.3390/ijms24087529 (PMC10145300; doi:10.3390/ijms24087529)

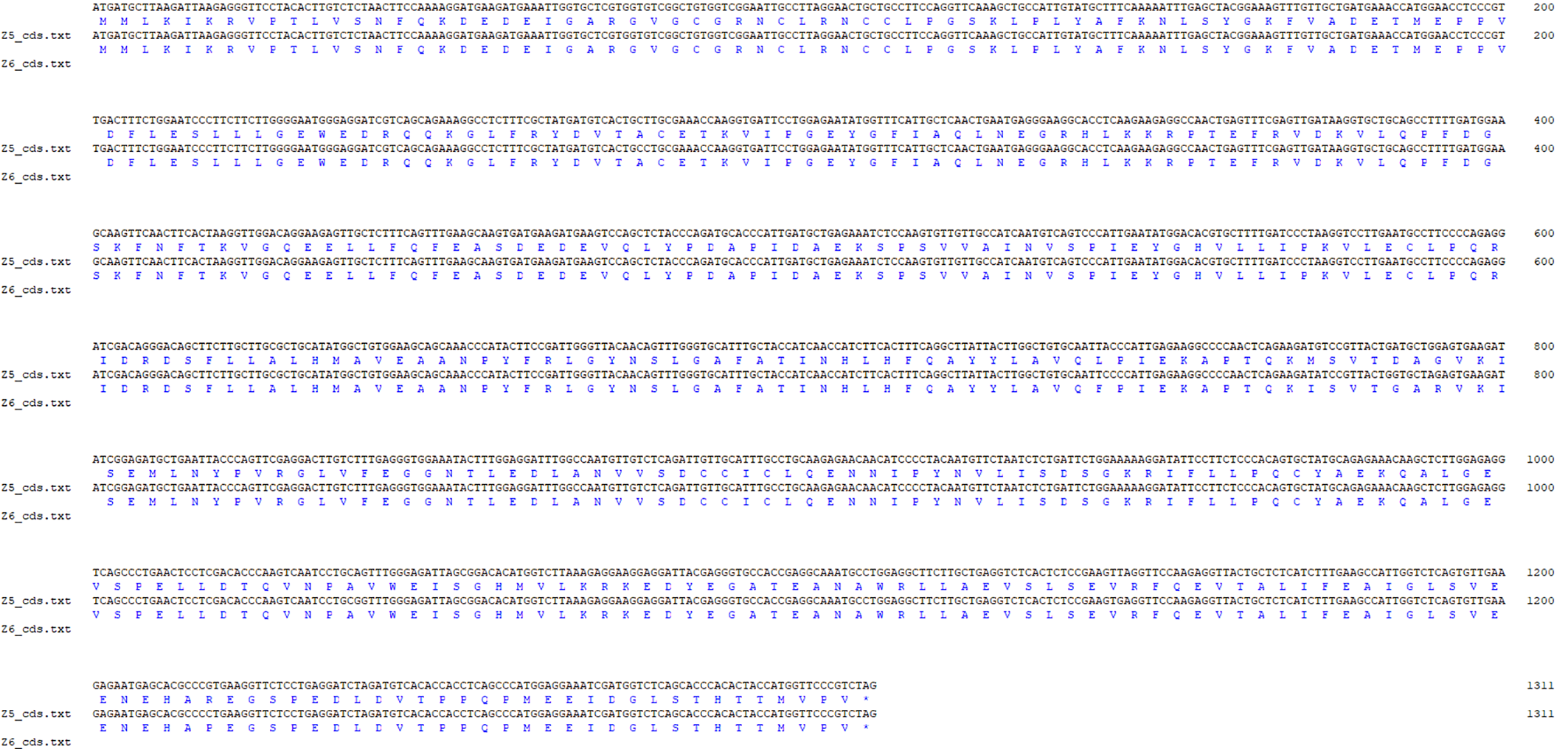

Supplement: Supplementary file 1 [file ijms-24-07529-s001.zip › Figure S1.tif]

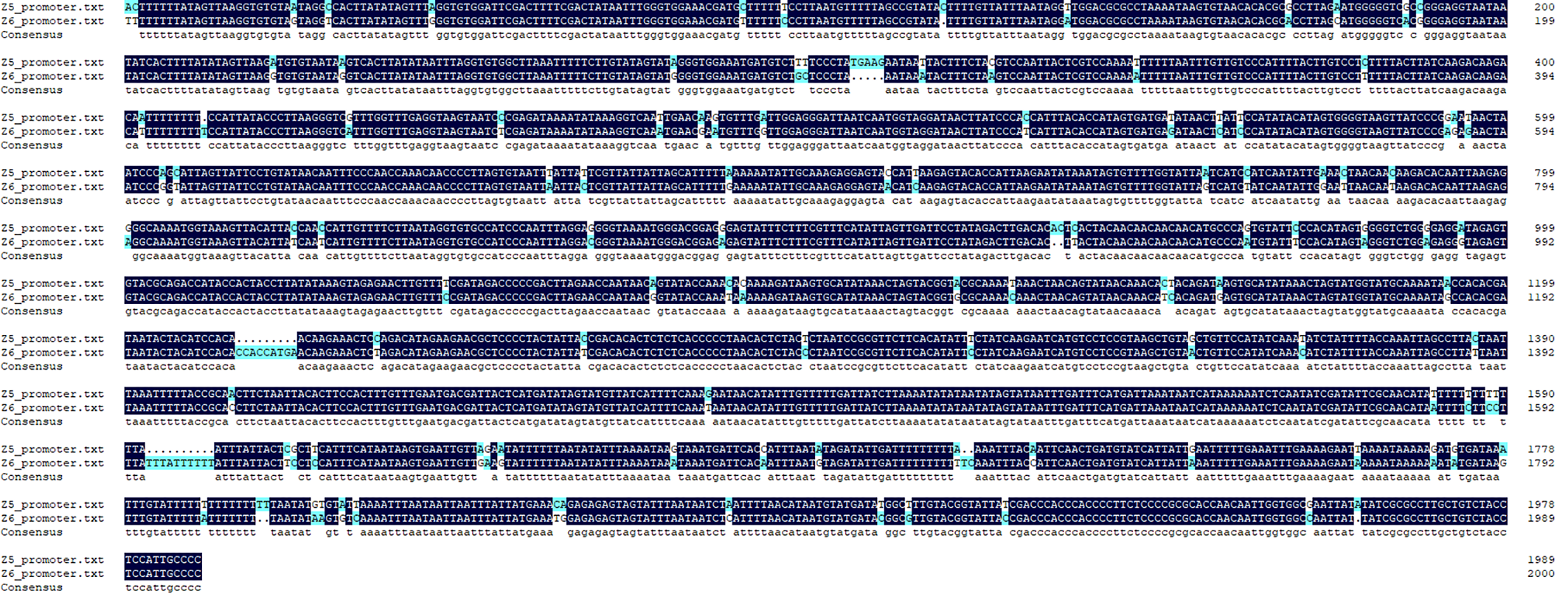

Supplement: Supplementary file 1 [file ijms-24-07529-s001.zip › Figure S2.tif]
